# Antithrombotic Treatment and Its Association with Outcome in a Multicenter Cohort of Transcatheter Edge-to-Edge Mitral Valve Repair Patients

**DOI:** 10.3390/jcdd9110366

**Published:** 2022-10-25

**Authors:** Christian Waechter, Felix Ausbuettel, Georgios Chatzis, Juan Cheko, Dieter Fischer, Holger Nef, Sebastian Barth, Philipp Halbfass, Thomas Deneke, Julian Mueller, Sebastian Kerber, Dimitar Divchev, Bernhard Schieffer, Ulrich Luesebrink

**Affiliations:** 1 Department of Cardiology, University Hospital Marburg, Baldingerstraße, 35043 Marburg, Germany; 2 Department of Cardiology, Cardiovascular Center Rotenburg/Fulda, Heinz-Meise-Straße 100, 36199 Rotenburg an der Fulda, Germany; 3 Department of Cardiology, University Hospital Giessen, Klinikstraße 33, 35392 Giessen, Germany; 4 Department of Cardiology, Cardiovascular Center Bad Neustadt/Saale, Von-Guttenberg-Straße 11, 97616 Bad Neustadt an der Saale, Germany

**Keywords:** MitraClip, anticoagulation, antiplatelet therapy, antithrombotics, mortality, outcome

## Abstract

Transcatheter edge-to-edge mitral valve repair (TEER) has become established as a safe and efficacious therapy for severe mitral regurgitation (MR) in high-risk patients. Despite its widespread use, postprocedural antithrombotic therapy (ATT) still to date is based on local expertise rather than evidence. In a multicenter, observational cohort study, 646 consecutive patients undergoing TEER were enrolled; 609 patients were successfully treated and antithrombotic therapy analyzed; 449 patients (73.7%) were previously treated with oral anticoagulants (OAC) due to the high prevalence of atrial fibrillation (459/609, 75.4%). Postprocedural ATT in patients previously treated with OAC consisted of no additional, additional single (SAPT) or dual antiplatelet therapy (DAPT) in 146/449 (33.6%), 248/449 (55.2%) and 55/449 (12.2%), respectively. There were 234/449 (52.1%) patients treated with vitamin-k antagonists (VKA) and 215/449 (47.9%) with nonvitamin-k antagonist oral anticoagulants (NOAC). One hundred sixty patients (26.3%) had no prior indication for OAC and were predominantly treated with DAPT (132/160, 82.5%). Use of SAPT (17/160, 10.6%) and no APT (11/160, 6.9%) was marginal. No statistically significant differences in terms of in-hospital mortality or the rate of major adverse cardiac and cerebrovascular events (MACCE) between the different antithrombotic therapy regimens were observed. Multiple Cox regression analysis showed a statistically significant decreased risk for all-cause mortality after a median follow-up of 419 days for OAC monotherapy (HR 0.6, 95%-CI 0.5–0.9, *p* = 0.04). This study provides evidence for a more favorable long-term outcome of OAC monotherapy in patients with an indication for OAC and reiterates the urgent need for randomized controlled trials on the optimal antithrombotic treatment of TEER patients.

## 1. Introduction

During the past decade, transcatheter edge-to-edge mitral valve repair (TEER) using the MitraClip^®^ device (Abbott Vascular, Santa Clara, CA, USA) has emerged as a major treatment modality for severe mitral regurgitation (MR) in a patient population that is not eligible for surgery. However, despite its use to date in more than 100,000 patients worldwide [1], postprocedural antithrombotic treatment is largely based on empiricism rather than evidence. This is particularly apparent in the lack of appropriate recommendations in the relevant guidelines (2017 ESC/EACTS, 2020 ACC/AHA) and in the markedly different antithrombotic strategies in randomized controlled trials [2,3]. For example, the antithrombotic regimen for patients in sinus rhythm varied from acetylsalicylic acid (ASA) 325 mg/d for six months in combination with clopidogrel 75 mg/d for 30 days in the EVEREST (Endovascular Valve Edge-to-Edge Repair Study) II trial to ASA 81 mg/d and/or clopidogrel 75 mg/d for at least six months in the COAPT (Cardiovascular Outcome Assessment of the MitraClip Percutaneous Therapy For Heart Failure Patients With Functional Mitral Regurgitation) trial [4,5]. In this context, the antithrombotic therapy becomes even more multifaceted when considering patients with an indication for additional oral anticoagulation. Based on a prevalence of coexistent atrial fibrillation (AF) of up to 73.3% reported from “real-world” data, this is the case in the majority of patients undergoing TEER [6,7,8,9]. In the absence of evidence-based recommendations, it seems to be appropriate to adopt the strategies used in patients undergoing surgical mitral valve repair (SMVR). Accordingly, postoperative transient oral anticoagulation (OAC) is generally recommended in the ESC/EACTS and ACC/AHA guidelines with a class IIa indication [2,3]. However, since TEER patients are older, more frail and exhibit more relevant comorbidities than surgically treated patients, this extrapolation has substantial limitations [10]. Furthermore, there is uncertainty about the comparability of surgical procedures using extracorporeal circulation with percutaneous intervention in terms of the associated prothrombotic risk. In sum, this highlights the need for an antithrombotic strategy specially tailored to the unique characteristics of the TEER collective. Indeed, safe and effective antithrombotic therapy constitutes a key aspect that will further improve the prognosis of this growing patient population. Addressing this issue, the present study aims to shed light on the current use of antithrombotic therapy regimens and their prognostic implications in a “real-world” multicenter collective of TEER. 

## 2. Methods

All consecutive patients at four German tertiary cardiac centers in whom TEER was planned between October 2011 and December 2021 were identified. Details on patient selection and procedural aspects have recently been published [11]. In the context of the present study, it should be noted all patients received unfractionated heparin during the procedure with a target ACT of 250–300 s. Periprocedural bridging with heparins in patients on VKA was attempted to be avoided, especially in the second half of the study period. In patients under anticoagulation with NOACs, the dose was paused on the intervention day and, in the absence of bleeding complications, NOAC therapy was continued on the subsequent day.

In the present retrospective observational cohort study, detailed medical characteristics, procedural parameters and especially medication were recorded. Particular emphasis was placed on the collection of antithrombotic medication and the duration of its prescription after TEER. Furthermore, changes in antithrombotic therapy prescribing patterns during the study period were recorded and stratified by preprocedural indication for oral anticoagulation. The corresponding data were collected in registries at each center and subsequently pooled for analysis.

Patients were followed up at regular intervals in the individual outpatient clinics of the participating heart centers, and if follow-up data were insufficient, they were supplemented by a survival query to the registry office for patients lost to follow-up. Despite the efforts made, 35 patients (5.7%) were lost to follow-up during the reported study period because of an unreported change in residence. However, there was no evidence of informative missingness and no significant impact of “lost to follow-up” patients on the results presented. The primary end points of the study were all-cause in-hospital death and all-cause mortality during follow-up, and the secondary end points were major adverse cardiac and cerebrovascular events (MACCE) during the index hospitalization. MACCE were defined as the occurrence of a cerebral and/or systemic thromboembolic event, a hemorrhage requiring intervention and/or transfusion or in-hospital death from a cardiovascular cause.

All statistical analyses were performed using R Studio V3.6.1 (R Foundation for Statistical Computing, Vienna, Austria), including the “survival”, “survminer”, “dplyr”, “networkD3”, “ggplot2” and “My.Stepwise” packages, and GraphPad Prism 6.0 (GraphPad Software, La Jolla, CA, USA). For categorical variables, data are presented as frequencies and percentages (%); for continuous variables, mean and standard deviation are presented for standard distributed variables, and median and interquartile range (IQR; 25th–75th percentile) are presented for nonstandard distributed variables. A two-sided *p* value of <0.05 was considered statistically significant. Differences between two groups were compared using the chi-square test and Fisher’s exact test for categorical variables, the t-test for standard distributed variables, and the Wilcoxon rank sum test for nonstandard distributed variables. Time-to-event analyses were carried out by using Cox regression. For adjustment, statistically significant predictors of all-cause mortality were identified using univariate Cox regression analysis and included in a multiple Cox regression model. Details of the included parameters are provided in the Results section. 

## 3. Results

### 3.1. Pre- and Postprocedural Antithrombotic Therapy and Prescription Duration

During the study period, 646 consecutive patients undergoing TEER with the MitraClip^®^ device were identified at the four participating heart centers. Thirty-seven patients underwent conservative treatment or surgical intervention after all due to insufficient reduction in MR severity and were therefore excluded from further analysis. None of the aborted procedures were related to bleeding or thromboembolic complications during the procedure. 

**Figure 1:**
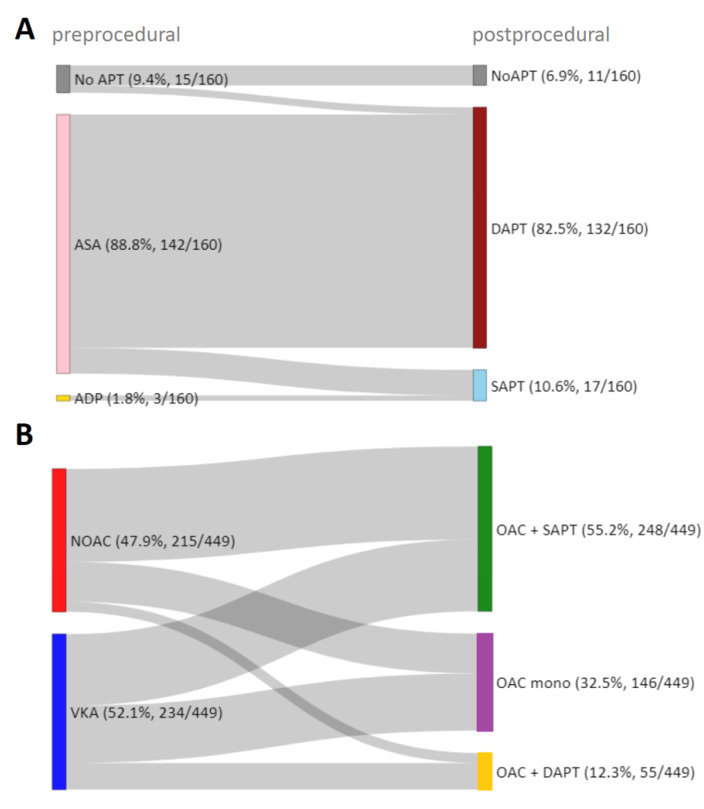
**Pre- and postprocedural antithrombotic therapy.** Sankey diagram illustrating the pre- and postprocedural antithrombotic therapy in successfully treated TEER patients without (**A**) and with (**B**) prior indication for oral anticoagulation. APT—antiplatelet therapy, ASA—acetylsalicylic acid, ADP—Adenosin-diphosphate receptor antagonists, SAPT—single antiplatelet therapy, DAPT—dual antiplatelet therapy, NOAC—nonvitamin k antagonist oral anticoagulant, VKA—vitamin k antagonist, OAC—oral anticoagulant.

Figure 1 shows the distribution of antithrombotic therapies before and after successful TEER, and Table 1 provides the baseline characteristics of the corresponding patients after stratification of postprocedural antithrombotic therapy. Patients who were already pretreated with acetylsalicylic acid (ASA; 142/609, 23.3%) or oral anticoagulation (OAC; 449/609, 73.7%) additionally received an antiplatelet agent after the procedure in the majority of cases, which was exclusively clopidogrel. Thus, most patients were treated postprocedurally with a combination of an oral anticoagulant (OAC) and an antiplatelet agent (248/609, 40.7%), followed by OAC mono (146/609, 24.0%), dual antiplatelet therapy (DAPT; 132/609, 21.7%), the combination of OAC and DAPT (55/609, 9.0%), and single antiplatelet therapy (SAPT; 17/609, 2.8%). Eleven patients (1.8%) did not receive antithrombotic treatment postprocedurally. DAPT consists of ASA and clopidogrel. Other antiplatelets such as prasugrel or ticagrelor were not used. Patients treated postprocedurally with oral anticoagulants despite no indication for OAC were not observed.

**Figure 2:**
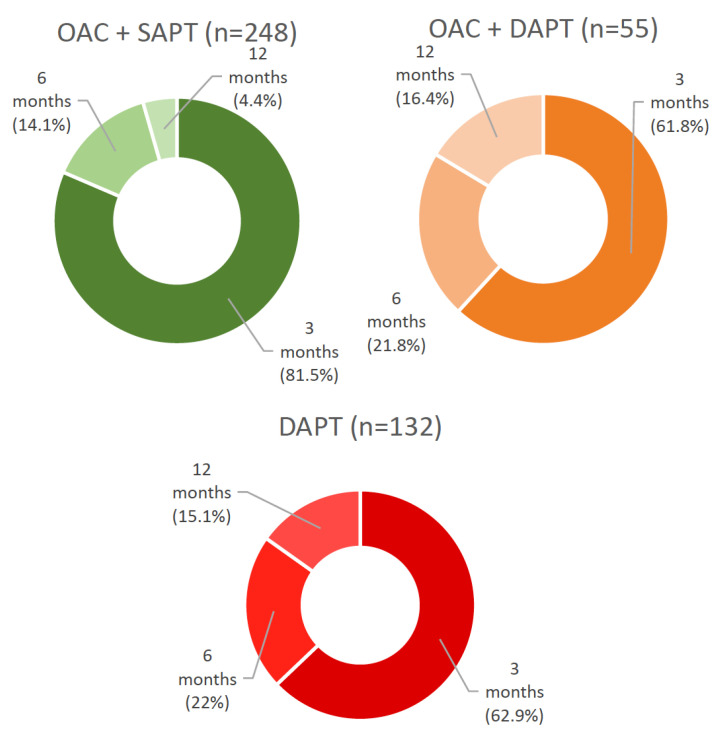
**Prescription duration of additional antiplatelet therapy after successful TEER.** OAC + SAPT—oral anticoagulant + single antiplatelet therapy, OAC + DAPT—oral anticoagulant + dual antiplatelet therapy, DAPT—dual antiplatelet therapy.

The prescription duration of additional postprocedural antithrombotics was further analyzed and is shown in Figure 2. In the studied population, additional antithrombotics were prescribed for 3, 6 or 12 months after the procedure, whereas in the majority of cases, antithrombotic therapy was intensified for three months after TEER (3-month duration in DAPT in 62.9%, in OAC + SAPT in 81.5%, in OAC + DAPT in 61.8% of cases). At the end of the indicated period, preprocedural treatment was resumed. 

### 3.2. Trends in Postprocedural Antithrombotic Therapy during the Study Period in Patients with an Indication for Oral Anticoagulation

**Figure 3:**
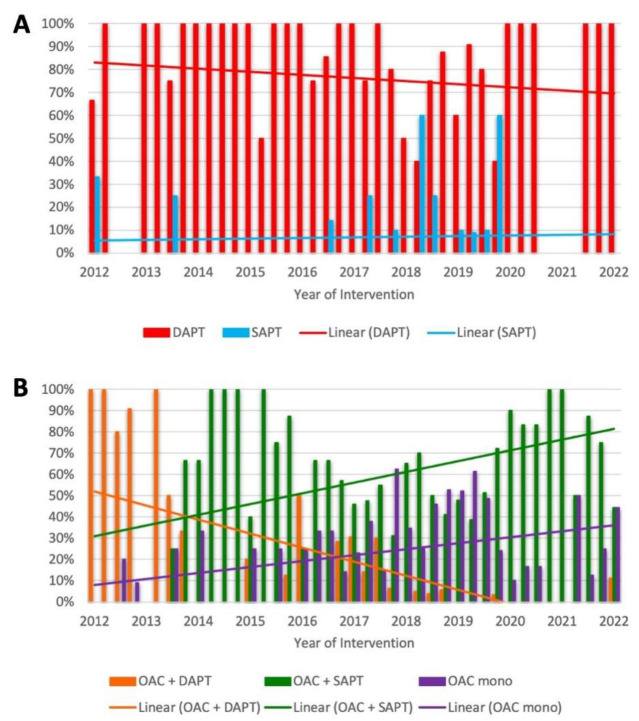
**Trends in postprocedural antithrombotic regimens during the study period in patients without (A) and with (B) an indication for oral anticoagulation.** (**A**) Quarterly plot with linear trend line of postprocedural prescription of single antiplatelet therapy (SAPT, blue) and dual antiplatelet therapy (DAPT, red). The percentages refer to all patients without indication for oral anticoagulation (OAC, 160/609). Comparison of prescriptions in first and second half of the study period: SAPT: 7.1% vs. 14.5%, *p* = 0.1; DAPT: 86.9% vs. 77.6%, *p* = 0.1. (**B**) Quarterly plot with linear trend line of postprocedural prescription of an oral anticoagulant (OAC mono, purple), a combination of OAC and SAPT (green) and a combination of OAC and DAPT (purple). Percentages refer to all patients with indication for OAC (449/609). Comparison of prescriptions in first and second half of the study period: OAC mono: 23.1% vs. 40.0%, *p* = 0.0001; OAC + SAPT: 51.8% vs. 58.0%, *p* = 0.2; OAC + DAPT: 25.1% vs. 2%, *p* < 0.0001.

The prescription patterns of antithrombotic therapy following TEER through time are shown in Figure 3. Postprocedural use of SAPT or DAPT in patients without an indication for OAC showed consistency during the study period. No statistically significant changes in the frequency of use in the first compared to the second half of the study period were observed (SAPT: 7.1% vs. 14.5%, *p* = 0.1; DAPT: 86.9% vs. 77.6%, *p* = 0.1). In contrast, there is a statistically significant decrease in the use of “triple therapy” (OAC + DAPT) in favor of an increase in the use of OAC monotherapy in patients with an indication for OAC when comparing the first and the second half of the study period (OAC mono: 23.1% vs. 40%, *p* = 0.0001; OAC + DAPT: 25.1% vs. 2%, *p* ≤ 0.0001). No statistically significant changes were observed with respect to the frequency of use of the combination OAC + SAPT in the first versus the second half of the study period (OAC + SAPT: 51.8% vs. 58.0%, *p* = 0.2).

### 3.3. Trends in VKA and NOAC Use during the Study Period

**Figure 4:**
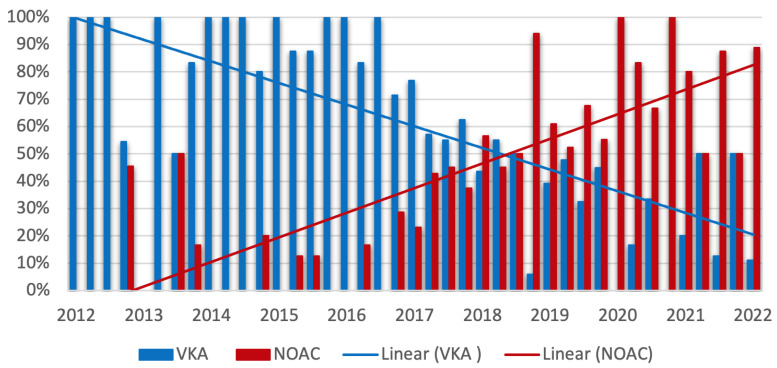
**Trends in prescription of vitamin K antagonists (VKA) and nonvitamin k antagonist oral anticoagulants (NOAC) during the study period**. Quarterly plot with linear trend line of prescription of VKA (blue) and NOAC (red). The percentages refer to all patients with indication for oral anticoagulation (449/609). Comparison of prescriptions in the first and second half of the study period: VKA: 72.9% vs. 35.6%, *p* < 0.0001; NOAC: 27.1% vs. 64.4%, *p* < 0.0001.

Figure 4 visualizes the change in the prescription of VKA and NOAC during the study period. Comparing the first half with the second half of the study period, a statistically significant decrease in the frequency of prescription of VKA (72.9% vs. 35.6%, *p* ≤ 0.0001) in favor of an increase in the use of NOAC (27.1% vs. 64.4%, *p* < 0.0001) was observed.

### 3.4. Association of Antithrombotic Therapy and Outcome

Table 2 presents major adverse cardiac and cerebrovascular events stratified by postprocedural antithrombotic treatment during the index hospitalization. Owing to the very small sample size, patients with postprocedural SAPT (17/609) and patients without any antithrombotics (11/609) were statistically not considered. No statistically significant differences were observed during the index hospitalization with respect to safety and efficacy in bleeding and transfusion rates, systemic or cerebral thromboembolism, or intrahospital cardiac or all-cause mortality as a function of anticoagulant or antiplatelet treatment.

Parameters statistically significantly related to long-term all-cause mortality were identified (male sex, chronic obstructive pulmonary disease, high grade tricuspid regurgitation, prior stroke, glomerular filtration rate, prior cardiac resynchronization therapy) and included in a multiple Cox regression model. This model showed that in patients with an indication for OAC, OAC monotherapy was associated with a statistically significant lower risk of death from any cause (hazard ratio (HR) 0.7, 95% confidence interval (CI) 0.5–0.9, *p* = 0.04) after a median follow-up duration of 419 days (IQR 658 days). The remaining antithrombotic regimens showed no statistically significant association with all-cause mortality even in a univariate Cox regression analysis. Details of the parameters of the multiple Cox regression model and the results of the univariate Cox regression of the other antithrombotic regimens are provided in Supplementary Tables S1 and S2.

## 4. Discussion

We provide a detailed analysis of antithrombotic therapy regimens and their impact on short- and long-term outcome in a well-characterized multicenter “real-world” collective of TEER patients using the MitraClip^®^ device. Owing to the high prevalence of coexisting atrial fibrillation (75.4%), the majority of patients received postprocedural antithrombotic therapy consisting of an oral anticoagulant (73.7%, 449/609). Overall, VKAs were used most frequently compared with NOACs (234/449, 52.1%, 215/449, 47.9%, respectively), and their prescription frequency decreased in favor of NOACs, especially since year 2017. Postprocedurally, in patients with indication for OAC, the combination of OAC + SAPT was used most frequently (248/449, 55.2%), followed by OAC mono (146/449, 32.6%) and OAC + DAPT (55/449, 12.2%). Patients without an indication for OAC (160/609, 26.3%) overwhelmingly received ASA preprocedurally (142/160, 88.7%) and were predominantly switched to DAPT with the addition of clopidogrel after TEER (132/160, 82.5%). Postprocedural SAPT with ASA or ADP antagonists (17/160, 10.6%) and no antithrombotic therapy (11/160, 6.9%) were negligible. Modification of antithrombotic therapy was prescribed over a three-month period in the majority of all reported combinations.

After stratification for postprocedural antithrombotic regimen, no statistically significant differences could be demonstrated for the rate of MACCE, cardiovascular mortality and all-cause mortality during the index hospitalization. When long-term prognosis was considered, patients with an indication for OAC showed a statistically significant lower risk of all-cause mortality with postprocedural OAC monotherapy (HR 0.6, 95%-CI 0.5–0.9, *p* = 0.04) during the median follow-up period of 419 days (IQR 658 days). No statistically significant effects on long-term outcome could be demonstrated for the remaining postprocedural antithrombotic therapies when stratified by prior indication for OAC.

As mentioned above, due to the lack of evidence-based data, antithrombotic treatment of TEER patients is empirical and therefore presents highly heterogeneous both in the “real-world” setting and in randomized controlled trails. Analogous to the extrapolation of experience from interventional closure of atrial septal defects, DAPT after TEER is most commonly used in patients with sinus rhythm. Knowing this background, and in agreement with reports from other registries and observational studies [12,13,14], the collective presented was also treated with DAPT for a duration of mostly 3 months in the vast majority of cases, unless there was an indication for OAC preprocedurally. The proportion of these patients without an OAC indication who received SAPT or no antithrombotics after TEER was negligible (17/160 and 11/160, respectively) and was therefore excluded from the statistical considerations. Nevertheless, it is noteworthy that these patients without any antithrombotics seemed to be clinically considerably more critically ill, as implied by the very high euroSCORE II (median 41.0), high portion of NYHA class IV (63.6%), the very high NT-proBNP level (median 12.325 ng/mL) and ultimately the excessive MACCE rate and in-hospital mortality (36.4% and 63.6%, respectively). A study based on German health claim data also showed that patients without antithrombotics had the comparatively worst outcome after TEER. Thus, an all-cause mortality of 50.3% was reported within 30 days after the procedure, with a significantly higher proportion of patients without antithrombotics (21%) than in our collective [15]. 

Considering the high prevalence of AF in TEER patients antithrombotic regimens containing oral anticoagulants are of great clinical relevance. In addition to the 75.4% AF prevalence reported here, this is also underscored by data from registries such as EVEREST II REALISM and ACCESS-EU (A Two-Phase Observational Study of the MitraClip^®^ System in Europe), which report concomitant AF in 66.5% and 67.7% of TEER patients, respectively [9,12,16]. In this significant proportion of TEER patients, the combination of an oral anticoagulant with an antiplatelet agent, predominantly clopidogrel, has become empirically established and was also specified in the study protocols of EVEREST II and COAPT [4,5]. Likewise, in this cohort, the majority of patients with OAC indication had a combination of OAC and SAPT postprocedurally, thus confirming other “real-world” data of Hohmann et al. cited above [15]. The regimen of “triple therapy” (OAC + DAPT), which was frequently used in the first quarter of the study period, was recently abandoned, which may be explained by evidence derived from the field of coronary interventions [17,18]. In this line, increasing “de-escalation” of antithrombotic therapy was observed in the second half of the study period with a comparatively more frequent use of OAC monotherapy after TEER. According to the data presented, this appears to have a positive impact on long-term outcome, as OAC monotherapy has been shown to be associated with a statistically significant lower risk for all-cause mortality after adjusting the baseline confounder. As the present study only assessed all-cause mortality, the reasons for the lower mortality risk in these patients can only be speculated. In addition to procedural success, prevention of bleeding and thromboembolic events are the major determinants of mortality in this unique patient population. Data from “real-world” registries and meta-analyses showed significantly higher rates of major bleeding than of thromboembolic events, ranging within the first 30 days from 3.5 to 13.4% and 0.7 to 2.6%, respectively [12,14,19,20]. However, there are conflicting results regarding the association between major bleeding complications and mortality after TEER. Whereas in two single center collectives no association of major bleeding events with increased mortality at both 30 days and 1 year could be demonstrated, von Bardeleben et al. found it to be an independent predictor of mortality early after the procedure in a German registry of more than 13,000 implants [21,22,23]. Regarding late bleeding events, a single center study by Benito-González et al. also demonstrated an independent association of bleeding events with increased mortality after a median follow-up of 523 days. Here, it was also shown that bleeding events were independently associated with antithrombotic combination therapy containing oral anticoagulants and antiplatelets [24]. The results of meta-analyses of combined antiplatelet and anticoagulant treatment also support these findings. They show up to double the relative risk of major bleeding and no significant advantage in preventing thromboembolic events with the use of VKA and ASA compared with VKA monotherapy [25,26]. When OAC is combined with clopidogrel, as is usually the case in TEER patients, the risk of bleeding may be even higher [27].

More recent data clarifying this issue in relation to the use of NOACs are currently not available. However, applying the results of the landmark studies of NOACs, a lower risk of major bleeding can be assumed with the use of NOACs compared with VKA [28]. 

On the other side of the complication spectrum, a recent meta-analysis totaling > 28,000 patients demonstrated that TEER procedures were associated with low rates of periprocedural and mid-term stroke (0.9% and 2.4%, respectively) [20]. Based on data derived from patients after surgical valve replacement, the benefit of OAC combination therapy (additional SAPT or DAPT) in reducing thromboembolic events compared with OAC monotherapy remains questionable, with a concomitant significant increase in the rate of bleeding complications as detailed above [29,30].

For patients without indication for OAC, no significant association of antithrombotic therapy regimens with all-cause mortality could be demonstrated in the present multiple Cox regression model. However, the comparatively limited sample size of this patient group, the negligible postprocedural use of SAPT, and the nonuse of oral anticoagulants limit the overall conclusions that can be drawn for this particular cohort of patients in the present study. The results of two small observational registries provide tentative evidence for a potential benefit of antithrombotic regimens containing oral anticoagulants even in patients without a prior OAC indication. Geis et al. reported a significantly lower incidence of stroke within 30 days after TEER in 157 patients without prior indication for OAC who were treated with VKAs for at least 30 days after the procedure [31]. Comparable cohorts were patients on DAPT from the previously published collectives of the ACCESS-EU registry, the German Registry for Transcatheter Mitral Valve Interventions (TRAMI) and the EVEREST II trial. The very small sample size and rate of events significantly limit the conclusiveness of the study. In another observational study, a significantly lower combined end point consisting of all-cause mortality, stroke, myocardial infarction and rehospitalization for heart failure 30 days after TEER was demonstrated in 136 patients treated with low dose apixaban (2.5 mg twice per day) and ASA for 4 weeks compared with 118 patients treated with SAPT or DAPT [32]. The conclusions of this study are also substantially limited due to the small sample size and lack of randomization.

## 5. Limitations

As this is an observational cohort study, the results cannot demonstrate a causal relationship, and despite careful adjustment for baseline differences, the possibility of residual bias remains. Furthermore, the results presented are descriptive and not exploratory. Thus, we cannot reconstruct the reasons for deciding on individual antithrombotic strategies. We are unable to report the incidence of bleeding or thromboembolic events in long-term follow-up and the specific causes of long-term mortality because the relevant data on these are not fully available. Nevertheless, highly relevant clinical end points were reported with a very low lost-to-follow-up rate.

## 6. Conclusions

Our aim was to provide a detailed overview of antithrombotic therapy regimens in a well-characterized, multicenter “real-world” collective of more than 600 TEER patients and demonstrate that in the majority of patients—those with an indication for OAC—the omission of additional antiplatelets is associated with a more favorable long-term outcome. Overall, the present study reemphasizes the urgent need for randomized controlled trials on the optimal antithrombotic treatment of TEER patients. 

## Figures and Tables

**Table 1:** **Characteristics of patients after successful TEER stratified by postprocedural antithrombotic therapy.** APT—antiplatelet therapy, SAPT—single antiplatelet therapy, DAPT—dual antiplatelet therapy, OAC—oral anticoagulant, NYHA—New York Heart Association, COPD—chronic obstructive pulmonary disease, CAD—coronary artery disease, CAB-OP—coronary artery bypass surgery, PCI—percutaneous coronary intervention, ICD—implantable cardioverter defibrillator, CRT—cardiac resynchronization therapy, GFR—glomerular filtration rate, LVEF—left ventricular ejection fraction, TR—tricuspid valve regurgitation, MR—mitral valve regurgitation, ACE—angiotensin-converting enzyme, AT1—angiotensin-II-receptor 1, ARN—angiotensin-receptor-neprilysin, ASA—acetylsalicylic acid, VKA—vitamin k antagonists, NOAC—nonvitamin k antagonist oral anticoagulant; data presented in percentage; absolute numbers of respective patients are given in brackets. * Median + interquartile range. † Mean + standard deviation; # *p*-values refer to the comparison of DAPT, OAC mono, OAC + SAPT and OAC + DAPT. Owing to the small sample size, the “no APT” and “SAPT” groups were excluded from the statistical comparison of the groups, which is indicated by the gray font color. Bold *p*-values indicate *p*-values < 0.05.

	Overall (*n* = 609)	No APT (*n* = 11)	SAPT (*n* = 17)	DAPT (*n* = 132)	OAC Mono (*n* = 146)	OAC + SAPT (*n* = 248)	OAC + DAPT (*n* = 55)	*p* -Value ^#^
Age (years)	78.1 ± 8	72.5 ± 11	74.3 ± 9.8	78.5 ± 7.6	78.9 ± 7.5	78.4 ± 8	76.2 ± 7.9	0.2
Male sex	61% (373)	72.7% (8)	64.7% (11)	53.8% (71)	58.9% (86)	63.3% (157)	63% (40)	0.08
euroSCORE II *	17.0 (21.7)	41.0 (43.8)	20.1 (15)	17.0 (20.6)	19.1 (23.1)	13.0 (20.7)	22.7 (24.3)	**<0.0001**
STS Risk Score *	7.0 (8.6)	18.8 (35.3)	7.3 (5.7)	7.0 (7.4)	6.6 (9.8)	6.7 (7.9)	8.4 (9.9)	0.3
NYHA class I NYHA class II NYHA class III NYHA class IV	0.2% (1) 4% (24) 71.8% (437) 24.1% (147)	0% (0) 9.1% (1) 27.3% (3) 63.6% (7)	0% (0) 0% (0) 70.6% (12) 29.4% (5)	0% (0) 6.1% (8) 73.5% (97) 20.5% (27)	0% (0) 2.7% (4) 76.7% (112) 20.5% (61)	0.4% (1) 3.2% (8) 71.8% (178) 24.6% (61)	0% (0) 7.4% (2) 59.3% (16) 33.3% (9)	0.6
COPD	21.2% (129)	27.3% (3)	35.3% (6)	24.2% (32)	20.5% (32)	19.4% (48)	18.2% (10)	0.7
CAD	67.2% (409)	81.8% (9)	76.5% (13)	78.8% (104)	54.8% (80)	65.3% (162)	74.5% (41)	**<0.0001**
Prior CAB-OP	27.1% (165)	18.2% (2)	52.9% (9)	34.1% (45)	21.2% (31)	25.8% (64)	25.5% (14)	0.1
Prior PCI	58.8% (358)	81.8% (9)	70.6% (12)	66.7% (88)	51.4% (75)	57.7% (143)	56.4% (31)	0.07
Diabetes mellitus	33% (201)	27.3% (3)	35.3% (6)	34.8% (46)	31.5% (46)	31.5% (78)	40% (22)	0.6
Arterial hypertension	82.8% (504)	36.4% (4)	82.4% (14)	80.3% (106)	81.5% (119)	87.1% (216)	81.8% (45)	0.3
Prior Stroke	10.8% (66)	0% (0)	5.9% (1)	8.3% (11)	9.6% (14)	12.5% (31)	16.4% (9)	0.3
Pre-existing ICD	25.5% (155)	9.1% (1)	41.2% (7)	20.5% (27)	19.9% (29)	27% (67)	43.6% (24)	**0.003**
Pre-existing CRT	13.3% (81)	0% (0)	11.8% (2)	11.4% (15)	12.3% (18)	17.7% (44)	3.6% (2)	**0.03**
Atrial fibrillation	75.4% (459)	81.8% (9)	35.3% (6)	17.4% (23)	92.5% (135)	94.4% (234)	94.5% (52)	**<0.0001**
GFR (mL/Min) †	48.3 ± 21.6	55 ± 30	58.2 ± 21	50.6 ± 23	47.9 ± 21	46 ± 20.8	47.7 ± 20.7	0.4
NT-proBNP (ng/L) *	2620 (4863)	12325 (16208)	2265 (4752)	2479 (3768)	2992 (4515)	2455 (5028)	2536 (6767)	0.3
LVEF (%) †	40.3 ± 14.1	30 ± 10.8	36.5 ± 14.6	39 ± 14	42.8 ± 14.4	41 ± 14	35.9 ± 13.3	**<0.001**
TR grade III	18.9% (115)	18.2% (2)	23.5% (4)	12.9% (17)	17.8% (26)	21% (52)	25.5% (14)	0.1
Degenerative MR etiology Functional MR etiology Mixed MR etiology	25.9% (158) 62.4% (380) 11.7% (71)	0% (0) 100% (11) 0% (0)	11.8% (2) 76.5% (13) 11.8% (2)	23.5% (31) 64.4% (85) 12.1% (16)	34.9% (51) 58.2% (85) 6.8% (10)	26.6% (66) 58.1% (144) 15.3% (38)	14.5% (8) 76.4% (42) 9.1% (5)	**0.01**
Number of clips implanted †	1.6 ± 0.6	2.2 ± 0.6	1.6 ± 0.7	1.5 ± 0.6	1.7 ± 0.7	1.6 ± 0.6	1.6 ± 0.6	0.053
Periprocedual MR reduction † (Carpentier grade)	2.1 ± 0.6	1.9 ± 0.7	1.9 ± 0.9	∆2.1 ± 0.6	2.2 ± 0.6	∆2 ± 0.6	∆2.1 ± 0.6	**0.02**
**Heart Failure Medication**								
ACE-/AT1 Inhibitors	74.6% (454)	27.3% (3)	70.6% (12)	74.2% (98)	71.9% (105)	79.4% (197)	70.9% (39)	0.3
ARN Inhibitor	8.9% (54)	0% (0)	17.6% (3)	5.3% (7)	11.6% (17)	9.3% (23)	7.3% (4)	0.3
Beta Blockers	88.3% (538)	45.5% (5)	88.2% (15)	87.9% (116)	88.4% (129)	90.3% (224)	89.1% (49)	0.9
Loop diuretics	89.5% (545)	36.4% (4)	88.2% (15)	88.6% (117)	91.8% (134)	91.5% (227)	87.3% (48)	0.6
Thiazid diuretics	21.8% (133)	0% (0)	23.5% (4)	22.7% (30)	18.5% (27)	25.8% (64)	14.5% (8)	0.2
Aldosteron antagonists	49.6% (302)	27.3% (3)	58.8% (10)	50% (66)	47.3% (69)	52.8% (131)	41.8% (23)	0.4
**Postprocedural Antithrombotic Medication**								
ASA	33.0% (201/609)	0% (0)	82.4% (14/17)	100% (132/132)	0% (0)	0% (0)	100% (55/55)	-
Clopidogrel	71.9% (438/609)	0% (0)	17.6% (3/17)	100% (132/132)	0% (0)	100% (248/248)	100% (55/55)	-
Other antiplatelets	0% (0/0)	0% (0)	0% (0)	0% (0)	0% (0)	0% (0)	0% (0)	-
VKA	38.4% (234/609)	0% (0)	0% (0)	0% (0)	58.9% (86/146)	43.5% (108/248)	72.7% (40/55)	-
NOAC	35.3% (215/609)	0% (0)	0% (0)	0% (0)	41.1% (60/146)	56.5% (140/248)	27.3% (15/55)	-

**Table 2:** **Rate of major cardiovascular and cerebrovascular events (MACCE) and all-cause in-hospital mortality during index hospitalization stratified by postprocedural antithrombotic therapy.** APT—antiplatelet therapy, SAPT—single antiplatelet therapy, DAPT—dual antiplatelet therapy, OAC—oral anticoagulant. Data presented in percentage; absolute numbers of respective patients are given in brackets. * *p*-values refer to the comparison of DAPT, OAC mono, OAC + SAPT and OAC + DAPT. Owing to the small sample size, the “no APT” and “SAPT” groups were excluded from the statistical comparison of the groups, which is indicated by the gray font color.

	Overall (*n* = 609)	No APT (*n* = 11)	SAPT (*n* = 17)	DAPT (*n* = 132)	OAC Mono (*n* = 146)	OAC + SAPT (*n* = 248)	OAC + DAPT (*n* = 55)	*p*-Value *
**Overall-MACCE** Cerebral/systemic thromboembolic event Bleeding requiring intervention In-hospital death from cardiovasc. cause	4.6% (28) 0.5% (3) 2.6% (16) 1.97% (12)	36.4% (4) 9.1% (1) 9.1% (1) 27.3% (3)	0% (0) 0% (0) 0% (0) 0% (0)	6.1% (8) 0% (0) 3.8% (5) 2.3% (3)	3.4% (5) 0.7% (1) 2.1% (3) 1.4% (3)	4% (10) 0.4% (1) 2.4% (6) 1.6% (4)	1.8% (1) 0% (0) 1.8% (1) 0% (0)	0.6 1 0.8 0.9
In-hospital death from any cause	3.94% (24)	63.6% (7)	11.8% (2)	3% (4)	2.7% (4)	2% (5)	3.6% (2)	0.8

## Data Availability

The data presented in this study are available upon request from the corresponding author. The data are not publicly available due to ethical restrictions.

## Supplementary Materials

The following supporting information can be downloaded at: https://www.mdpi.com/article/10.3390/jcdd9110366/s1, Table S1: Independent predictors of all-cause mortality of patients without prior indication for OAC in a univariable Cox regression model; Table S2: Independent predictors of all-cause mortality of patients with preprocedural indication for OAC in a univariable Cox regression model.
