# Supplementary material for: Antithrombotic Treatment and Its Association with Outcome in a Multicenter Cohort of Transcatheter Edge-to-Edge Mitral Valve Repair Patients
Source: J Cardiovasc Dev Dis. 2022 Oct 25;9(11):366. doi: 10.3390/jcdd9110366 (PMC9695441; doi:10.3390/jcdd9110366)
Supplement: Supplementary file 1 [file jcdd-09-00366-s001.zip › jcdd-1967233-supplementary.pdf]

Variable	Hazard Ratio	95%-CI	p-value
Male sex	2.2	1.2-4.4	0.02
COPD	1.9	1.008-3.4	0.047
TR grade III	3.3	1.7-6.5	<0.001
Stroke	2.0	0.9-4.3	0.07
GFR >60mL/Min	0.5	0.2-0.9	0.03
GFR <30mL/Min	2.0	1.005-3.9	0.048

Table S1: Independent predictors of all-cause mortality of patients without prior indication for OAC (n = 160) in a univariable Cox regression model.

Variable	Hazard Ratio	95%-CI	p-value
OAC mono	0.6	0.5-0.9	0.02
Male sex	1.4	1.02-1.9	0.04
Age (65-75ys)	0.7	0.5-1.03	0.07
Age (<65ys)	1.5	0.9-2.6	0.12
COPD	1.4	1.01-1.9	0.04
CRT	1.5	1.05-2.3	0.03
TR grade III	1.6	1.2-2.6	0.003

Table S2: Independent predictors of all-cause mortality of patients with preprocedural indication for OAC (n = 449) in a univariable Cox regression model**.**
